# Frequency-dependent drug screening using optogenetic stimulation of human iPSC-derived cardiomyocytes

**DOI:** 10.1038/s41598-017-09760-7

**Published:** 2017-08-29

**Authors:** Hendrik Lapp, Tobias Bruegmann, Daniela Malan, Stephanie Friedrichs, Carsten Kilgus, Alexandra Heidsieck, Philipp Sasse

**Affiliations:** 1 Institute of Physiology I, Life and Brain Center, Medical Faculty, University of Bonn, Sigmund-Freud-Str. 25, 53127 Bonn, Germany; 2 Research Training Group 1873, University of Bonn, 53127 Bonn, Germany; 3 Zentralinstitut für Medizintechnik, Technische Universität München, München, Germany

## Abstract

Side effects on cardiac ion channels are one major reason for new drugs to fail during preclinical evaluation. Herein we propose a simple optogenetic screening tool measuring extracellular field potentials (FP) from paced cardiomyocytes to identify drug effects over the whole physiological heart range, which is essential given the rate-dependency of ion channel function and drug action. Human induced pluripotent stem cell-derived cardiomyocytes were transduced with an adeno-associated virus to express Channelrhodopsin2 and plated on micro-electrode arrays. Global pulsed illumination (470 nm, 1 ms, 0.9 mW/mm^2^) was applied at frequencies from 1 to 2.5 Hz, which evoked FP simultaneously in all cardiomyocytes. This synchronized activation allowed averaging of FP from all electrodes resulting in one robust FP signal for analysis. Field potential duration (FPD) was ~25% shorter at 2.5 Hz compared to 1 Hz. Inhibition of hERG channels prolonged FPD only at low heart rates whereas Ca^2+^ channel block shortened FPD at all heart rates. Optogenetic pacing also allowed analysis of the maximum downstroke velocity of the FP to detect drug effects on Na^+^ channel availability. In principle, the presented method is well scalable for high content cardiac toxicity screening or personalized medicine for inherited cardiac channelopathies.

## Introduction

The cardiac action potential (AP) is formed by sequential opening and closing of specific ion channels. In a human ventricular cardiomyocyte, the initial fast depolarization is carried by Na^+^ currents from SCN5a (Nav1.5) channels and this is essential for the fast propagation of electrical activity throughout the heart. Subsequently the AP plateau is sustained by opening of L-type Ca^2+^ channels and the resulting influx of Ca^2+^ triggers Ca^2+^ release from the intracellular stores to initiate contraction. The human AP is terminated by closing of Ca^2+^ channels and activation of two repolarizing potassium conductances: a rapid activating delayed rectifier current (I_Kr_) through hERG channels and a slow activating component (I_Ks_) by KvLQT channels which are redundant and the basis for the “repolarization reserve”^1^. The resting membrane potential between APs is ensured by inward rectifying potassium currents (I_K1_) through Kir2.1 channels^2^.

Inherited ion channel mutations, side effects of drugs or electrolyte imbalance can affect the activity of these channels, thereby changing the shape of APs with a high risk of potential lethal ventricular tachyarrhythmia. One of the most common cardiotoxic effects of drugs is delayed cardiac repolarization resulting in prolonged APs, which clinically manifests in the electrocardiogram as a long QT interval and is therefore called long QT-syndrome (LQTS). Further highly pro-arrhythmic drug-induced conditions are the short QT syndrome (SQTS) caused by side effects on Ca^2+^ channels and the Brugada syndrome (BS) due to block of Na^+^ channels or dispersion of early repolarization^3, 4^.

In the last 30 years, side effect of drugs on cardiac ion channels were one major cause for drug withdrawals from the market. Currently, the FDA requires screenings for cardiac safety with cell lines overexpressing the hERG K^+^-channel, because this channel is responsible for most of the drug effects on cardiac repolarization^5–7^. However, the electrical activity of cardiomyocytes and generation of APs are regulated by many other Na^+^, Ca^2+^ or K^+^ channels that are not present in such heterologous expression systems which is why screening of drug effects in cardiomyocytes would be beneficial^8^. Thus, the identification of novel suited screening systems became a central paradigm in the FDA “Comprehensive *in-vitro* Proarrhythmia Assay” (CiPA) initiative^7^ to assure patients’ safety and to save billions of dollars that are lost because of late drug withdrawals^6, 9, 10^. Specifically the CiPA initiative aims to identify multiple comprehensive and multifaceted methods whose results can be integrated to permit a more direct assessment of whether a drug has a propensity to cause cardiac arrhythmias^7, 11^.

Unfortunately cardiomyocytes from animals have distinct differences to human cardiomyocytes in ion channel composition^12, 13^ and sufficient amount of primary human cardiomyocytes for screening purposes cannot be obtained by biopsies. Cardiomyocytes differentiated from human embryonic stem cells or from induced-pluripotent stem cells (hiPSC) were identified as a novel option for drug screening because their ion channel expression is similar to primary human cardiomyocytes^14, 15^.

The gold standard for investigating electrical properties of cardiomyocytes is the patch clamp technique. However, this is very time consuming and the duration of experiments is limited due to run-down of cytosolic stability^16^. Recent studies demonstrated the use of optical voltage and intracellular Ca^2+^ imaging and suggested the applicability for high-throughput cardiac toxicity screening assays^17–19^ with the advantage of optical mapping technologies allowing in principle single cell measurements within a cardiac syncytium.

Although these optical read-out systems provide important data on AP and Ca^2+^ transient duration, they are based on rather complex, custom-built and cost-intensive instrumentation that is commercially not available. Furthermore genetically encoded voltage indicators^19^ require very high intensity laser light illumination and voltage sensitive dyes must be loaded into the cell^17, 18^, which both could be toxic to cells and do not allow longitudinal studies of long term drug effects.

The analysis of the electrical activity by recording the extracellular field potentials (FP) with commercially available microelectrode arrays (MEA) recording systems is a well-suited, scalable and non-optical alternative for analysis of cardiac toxicity^20^. This technique has been already proven to be effective in determining drug effects on cardiac ion channels because the duration of FP (FPD) is highly correlated to the AP duration (APD)^21^ and it gathered importance since its development^1, 6, 10, 20, 22–25^. Furthermore, initial rapid fast component of the FP reflects the fast upstroke at the beginning of the AP^21^ and could potentially be used to determine Na^+^ channel availability. The specific advantages of this technology are the easy-to-use commercially available instrumentation and the lack of dye loading or high intensity illumination, which enables the long-term analysis over days in the incubator. This could be an important aspect for drug screening assays as it was shown before that tyrosine kinase inhibitors used in cancer therapy and some I_Kr_ inhibitors show additional pro-arrhythmic effects on the late Na^+^ current only after long term application^26, 27^.

However, the current available MEA technology also has its limitations as FP shapes usually vary between the individual electrodes and thus suited electrodes are usually manually chosen by the investigator which prevents automatized analysis. In addition, electrical stimulation of cardiomyocytes on MEA generates recording artefacts, produces the toxic gases H_2_, O_2_ or Cl_2_ and alters pH due to faradaic reactions especially during long stimulation periods required for drug screening^28^. Furthermore, the large stimulation artefacts at the beginning of the FP signals makes the analysis of the initial rapid FP component impossible. This is why most studies of cardiomyocytes on MEA are done during spontaneous beating^1, 6, 10, 20, 22–25, 29, 30^. However drug effects on cardiac excitability and action potentials can depend on the heart rate because of use-dependent properties of cardiac ion channels and the rate-dependency of drugs^31–33^.

Here we report a novel technology for drug screening on human cardiomyocytes at the whole range of physiological heat rates using optogenetic pacing^34^ on MEA. This enables artefact-free and synchronized stimulation of cardiomyocytes with light pulses, which allows averaging of FP from the single recording electrodes for objective analysis. We validated the new approach by testing well-known drugs leading to LQTS, SQTS and BS at heart rates ranging from 60 to 150 bpm. Finally we provide evidence for magnetic positioning of hiPSC-derived cardiomyocytes selectively on MEA recording electrodes which in the future could be very useful for automatized screening assays with low cell numbers.

## Experimental Procedures

### Cell culture and generation of ChR2-expressing cardiomyocytes

1 × 10^6^ Cor4U cardiomyocytes derived from hiPSC were purchased from Axiogenesis (Cologne, Germany) and cultivated in Cor4U Complete Culture Medium (Axiogenesis) in T25 flasks. This cell line has been previously characterized in detail^17, 35, 36^ and consists of ventricular (~60%), atrial (~20%) and pacemaker-like (20%) cardiomyocytes^37^. For expression of ChR2, Cor4U cardiomyocytes were transduced in the T25 flask with an adeno-associated virus (AAV) at 6.6 × 10^4^ genome copy numbers per cell. We used a serotype 2 AAV with serotype 1 capsid for expressing ChR2 (H134R) fused to the red fluorescence protein mCherry under the control of the chicken-β-actin promotor (AAV2/1-CAG-hChR2(H134R)-mCherry), provided by Penn Vector Core (University of Pennsylvania). Two days after transduction, Cor4U cardiomyocytes were detached from the T25 flask and dissociated by 5 minutes incubation with 5 ml PBS and 2 µM EDTA at room temperature and subsequently 5 minutes incubation with 800 µl TripLE™ Express (Life Technologies) at 37 °C. Dissociated cells were incubated for 20 min in Cor4U Complete Culture Medium at 4 °C to recover, and then used for staining, FACS, patch clamp or MEA analysis.

### Determination of ChR2 expression

To quantify ChR2 expression, ChR2-transduced and dissociated Cor4U cardiomyocytes were replated on fibronectin-coated coverslips (10 µg/ml) and cultivated for 3 days in Cor4U culture medium. Percentage of mCherry expressing Cor4U cardiomyocytes was analysed with a CyFlow Space FACS machine (Partec GmbH, Münster, Germany, 561 nm laser excitation, 590 ± 50 nm emission). For immunohistochemical stainings, Cor4U cardiomyocytes were fixated with 4% PFA, permeabilized with 0.2% Triton X (Sigma-Aldrich) in PBS and stained in 5% donkey serum (Jackson ImmunoResearch) with primary antibodies against the protein α-actinin (1:400, Sigma-Aldrich) for 2 hours and a Cy2-conjugated secondary antibody (1:100, Jackson ImmunoResearch) in 0.1% Hoechst 33342 (Sigma-Aldrich) for 1 h. Pictures were taken with an inverted fluorescence microscope (Axiovert 200 M, Zeiss) equipped with the Apotome section module, through a 25x Plan-Apochromat objective (Zeiss), mCherry (F46–008), Cy2 (HC 470/22ex, HC 495bs, HC 510/10em) filter sets (all from AHF Analysentechnik) and the AxioCam MRm camera with the AxioVision software (Version 4.8.2, Zeiss).

### Patch Clamp experiments

ChR2-transduced and dissociated Cor4U cardiomyocytes were re-plated at low densities on fibronectin-coated (10 µg/ml) coverslips and investigated 48–72 h later in the whole cell configuration using an EPC10 amplifier (Heka) with an internal solution containing (in mmol/L) 50 KCl, 80 K-Aspartate, 1 MgCl_2_, 3 MgATP, 10 EGTA, 10 HEPES, pH 7.2 (KOH) and an external solution containing 140 NaCl, 5.4 KCl, 1.8 CaCl_2_, 1 MgCl_2_, 10 HEPES, 10 Glucose, pH 7.4 (NaOH) as reported previously^34, 38^. Cor4U cardiomyocytes were illuminated with blue light (470 nm) from a temperature-controlled LED module (LEDMOD LAB 470 nm, Omicron Laserage) coupled to the epifluorescence port of an Axiovert 200 microscope (Zeiss) and controlled by the EPC10 amplifier. Light-induced currents were characterized at a holding potential of −40 mV with supramaximal light stimulation of 5 mW/mm^2^ for 1000 ms. Peak currents were analysed by determining the maximal inward current and steady-state currents by averaging the currents between 300 to 900 ms of the light pulse using Fitmaster (Heka). APs were recorded in the current clamp mode and were elicited either by 2 ms long current injections through the patch pipette with increasing amplitude (from 0 to 1000 pA in 100 pA steps) or by 1 ms long blue light pulses (470 nm) at increasing light intensities (0–5 mW/mm^2^). Stimulation at each current amplitude and light intensity was repeated 5 times at a frequency of 1 Hz and the lowest stimulation which stably elicited APs was analysed. APD was calculated from the peak to 90% of repolarization (APD_90_) using the cardiac AP analysis module of the LabChart software (AD Instruments).

### MEA recordings

MEA with 59 electrodes of 30 μm diameter and 200 μm interelectrode spacing (60MEA200/30iR-Ti) were coated with a 2 µl drop of 10 μg/ml fibronectin (Sigma Aldrich) covering the field of electrodes, before 2 × 10^4^ ChR2-AAV transduced dissociated Cor4U cardiomyocytes were plated and cultured on MEA in Cor4U medium. Local FP were recorded with the MEA 1060 System and the MC Rack software (Multichannel Systems) at 10 kHz sampling rate 3–5 days after plating in 37 °C Tyrode’s external solution comprising 142 mM NaCl, 4.4 mM KCl, 2.0 mM MgCl_2_, 1.8 mM CaCl_2_, 10.0 mM Glucose, 10.0 mM HEPES (pH 7.4 NaOH).

The whole recording area (1.4 mm × 1.4 mm) of the MEA was illuminated with a 470 nm LED (LEDC5 and LEDD1, Thorlabs) controlled with the Powerlab 8/30 and the Chart 7.1 software (AD Instruments) and attached to the epifluorescence port of a MVX10 macroscope equipped with a 1x objective (MVPLAPO, numerical aperture: 0.25, Olympus). Light intensity was calibrated by measuring the light power with the PM100 powermeter and S130A sensor (Thorlabs) at the level of the MEA electrodes.

To determine the minimal light intensity required for stable pacing at a given pulse duration, trains of 15 light pulses (at 1.5 Hz) were applied with stepwise (~0.25 mW/mm^2^) increasing light intensity. The lowest light intensity at which each of the last 10 pulses induced a FP was defined the threshold for stable pacing. This protocol was repeated for 1, 2, 5, 10 and 25 ms pulse durations.

Effect of drugs on the FP were recorded in blocks of at least 20 s during continuous optogenetic pacing (1 ms pulses, 0.9 mW/mm^2^) at a fixed rate varying from 1 to 2.5 Hz. Only experiments with stable 1:1 pacing over the whole time of recording were taken into account. After baseline recording, drugs were added at the indicated concentrations to the Tyrode solution from following stock solutions: E4031: 10 mM dissolved in H_2_O, Sotalol: 10 mM dissolved in H_2_O, Quinidine: 10 mM dissolved in EtOH, Verapamil: 1 mM dissolved in H_2_O, JNJ303: 25 mM dissolved in DMSO, Lidocaine: 100 mM dissolved in EtOH.

### FP analysis

Color-coded activation maps with isochronal lines were generated using a custom written software (Labview, National Instruments) by calculating the local activation time of each electrode (minimum dV/dt) and interpolating electrodes with noisy signals. For a systematic analysis of FP shape, one temporally and spatially averaged FP per recording block was calculated. Therefore the recorded FP from each electrode were temporally averaged over the whole recording block with the average tool of the MC Rack software using as trigger the onset of illumination during optogenetic stimulation or one FP during spontaneous activity.

Next, electrodes were analysed for electrical activity (defined as >15 µV minimum negative deflection) and recording blocks with >4 active electrodes were further processed. In average, 31 ± 1 active electrodes out of the 59 total electrodes (n = 160 recording blocks) were spatially averaged by calculating the mean FP shape using the Origin Pro 8 G software (Version 8.0988, OriginLab). Of note, this spatial averaging was only possible because of the simultaneous activation without conduction delay using global optogenetic pacing (see results).

FPD was determined by measuring the time between the minimum of the FP (t_min_) and maximal value of the repolarization wave (t_end_). t_end_ was identified by FP smoothing (Savitzky-Golay filter, polynomial order 2, 600 samples window) with subsequently calculating the first derivative and identification of the time point during the repolarization wave where dV/dt = 0.

Maximum downstroke velocity (Max.DV) was calculated by the minimum value of the non-smoothed first derivative of the FP.

### Local targeting using magnetic nanoparticles (﻿MNP)

Cardiomyocytes were loaded and purified with SO-Mag5 MNP (50 pg iron/cell, SO-Mag5 stock contains 15.8 µg iron/µl) as reported before^39–41^. To enhance the uptake of the MNP in cells, MNP were preincubated with the DreamFect Gold Transfection Reagent (OZBiosciences) (0.25 µl/µg iron) in 200 µl H_2_O for 15 min at room temperature. Thereafter 1 × 10^6^ Cor4U cardiomyocytes were incubated with this solution overnight in Cor4U Complete Culture Medium in T25 flask. The next day, the cardiomyocytes were dissociated as described above, MNP loaded cardiomyocytes were purified in a magnetic rack as previously described^39, 41^. Finally, 1 × 10^4^ magnetic labeled cardiomyocytes were seeded into each well of a fibronectin (10 μg/ml) coated 6-well MEA in Cor4U medium. The MEA was placed on a custom-designed magnet setup to target the cardiomyocytes to the field of electrodes and incubated overnight at 37 °C in an incubator. The magnet setup consisted of 12 (one below and one above each well) permanent magnets with 1 mm diameter and 7 mm height made of NdFeB with N48 magnetization, which corresponds to a remanent flux density of approximately 1.38 T (Z01 × 07Ni-N48, HKCM Engineering e.K. Eckernfoerde, Germany). The resulting magnetic flux density and magnetic gradient were calculated using the software Comsol Multiphysics 5.1 (Comsol Multiphysics GmbH, Goettingen, Germany) as reported earlier^39^.

### Statistical analysis

All data are shown as mean ± s.e.m and absolute FPD values were taken for statistical analysis. Significance between FPD at various frequencies was analysed by 1-Way ANOVA tests using Dunnett’s multiple comparison test. APD_90_ values and effects of pharmacological substances on prolongation of FPD for each heart rate were analysed by two-tailed paired Student’s *t*-test. A *p* value of <0.05 was considered statistically significant. The ‘*n*’ values are indicating the number of independent experiments. The number of individual cell batches used for the statistics are indicated. Data were analysed and represented with Graph Pad Prism (Version 5.01, GraphPad Software).

## Results

### Optical stimulation of ChR2-expressing cardiomyocytes

**Figure 1:**
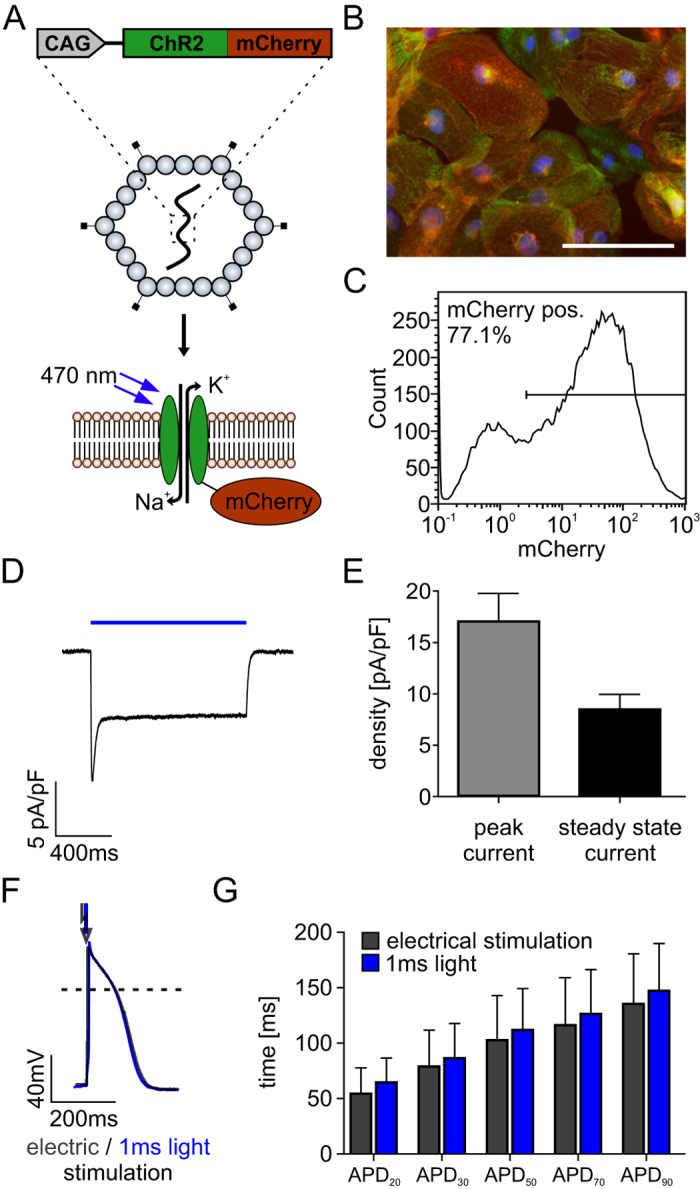
ChR2 expression and characterization in hiPSC derived cardiomyocytes. (**A**) Schema of the adeno-associated virus (AAV) construct for expression of ChR2 and mCherry under the control of the chicken β-actin (CAG) promoter (top) and illustration of ChR2, a light-induced non-selective cation channel (bottom). (**B**) mCherry signals (red) indicating ChR2 expression in α-actinin positive (green) cardiomyocytes (nuclear staining in blue, scale bar: 100 µm). (**C**) Representative example of FACS analysis of mCherry positive cardiomyocytes after AAV transduction. (**D**) Representative current trace during illumination (5 mW/mm^2^) at a holding potential of −40 mV. (**E**) Quantification of light-induced peak- and steady state photocurrents (n = 19 from 1 batch). (**F**) Overlay of APs induced by electrical (400 pA current injection, 2 ms, black) and optical stimulation (1.4 mW/mm^2^, 1 ms, blue). (**G**) Action potential duration at 20%, 30%, 50%, 70% and 90% of repolarization (APD) during electrical and optical pacing (APD20: p = 0.1995; APD30: p = 0.4027; APD50: p = 0.3064; APD70: p = 0.2647; APD90: p = 0.1893, n = 13 from 1 batch, two-tailed, paired student’s *t*-test).

We used commercially available, purified cardiomyocytes derived from hiPSC and inscribed light sensitivity by transduction with AAV for expressing the non-selective cation channel ChR2 (H134R)^42^ fused to the red fluorescence protein mCherry (Fig. 1A,B). FACS analysis revealed that 70.8 ± 9.6% (n = 3 from 3 cell batches) of cardiomyocytes expressed mCherry (Fig. 1C) and we did not detect negative effects on cell-viability. In these cells, illumination with blue light (470 nm, 5 mW/mm^2^) induced inward photocurrents typical for ChR2 H134R (Fig. 1D,E)^42, 43^. Light pulses as short as 1 ms were sufficient to evoke APs with characteristic properties of hiPSC-derived cardiomyocytes (Fig. 1F) and with identical APD to those evoked by current injection through the patch pipette (Fig. 1G) excluding an influence of photocurrents on the APD.

### Optogenetic stimulation on MEA

**Figure 2:**
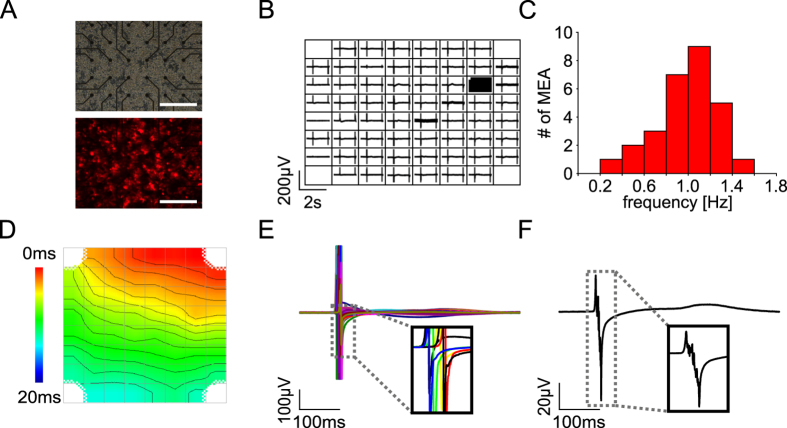
MEA recordings of spontaneously beating cardiac syncytia. (**A**) Bright field- (top) and fluorescence (bottom, red: mCherry) image of 2 × 10^4^ cardiomyocytes plated on a MEA. Scale bars: 400 µm. (**B**) Overview of FP recordings from the 59 MEA electrodes during spontaneous beating. (**C**) Histogram of spontaneous beating frequencies (n = 28 from 5 batches). (**D**) Local activation map during spontaneous beating (activation time color coded, isochronal lines 1 ms apart). (**E**) Overlay of FP during spontaneous activity; inset highlights the delay between FP from 6 electrodes. (**F**) Mean FP averaged from electrodes with signals during spontaneous activity.

To test the advantages of optogenetic stimulation of cardiomyocytes, we plated the AAV-transduced hiPSC-derived cardiomyocytes on MEA (Fig. 2A) on which they formed a spontaneously beating syncytium with ChR2 expressing and non-expressing cardiomyocytes equally distributed (Fig. 2B). At 37 °C, we observed beating frequencies ranging from 0.3 to 1.5 Hz (Fig. 2C). Latency analysis showed that electrical activity was initiated locally at one edge of the MEA and conducted to the rest of the syncytium (Fig. 2D) with an average conduction velocity of 9.5 ± 2.2 cm/s (n = 3, from 1 batch). Thus excitation of all cardiomyocytes on the 1.4 × 1.4 mm MEA takes about ~ 20 ms. Because of this sequential activation of cells (Fig. 2E), averaging of FP from all electrodes resulted in smearing and fragmentation of the initial fast component of the FP (Fig. 2F).

**Figure 3:**
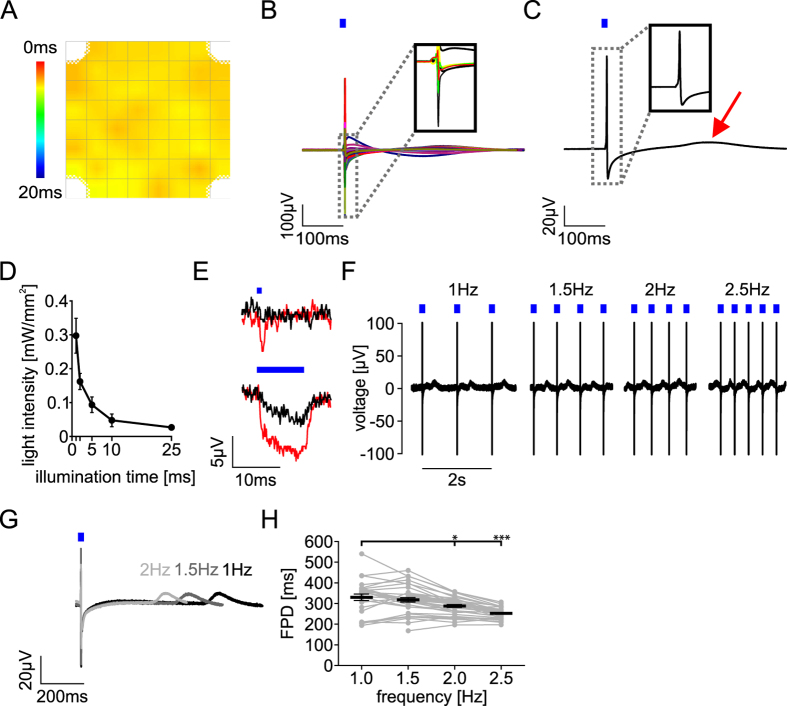
Optical stimulation of cardiomyocytes on MEA. (**A**) Local activation map during global optical pacing by illumination of all electrodes (activation times colour coded as in Fig. 2D). (**B**) Overlay of FP during optogenetic pacing; insert highlights FP from the 6 electrodes as in Fig. 2E. (**C**) Mean FP averaged from electrodes with signals during global optical pacing. Red arrow highlights the maximal value of the repolarization wave (t_end_). (**D**) Light intensity and duration relationship for stable optical pacing at 1.5 Hz (n = 4 from 1 batch). (**E**) Electrical signals from MEA electrodes without cells upon application of 1 ms (top) and 10 ms (bottom) light pulses of 0.9 mW/mm^2^ (black) and, 2.7 mW/mm^2^ (red). (**F**) Representative FP recording from one electrode upon optical stimulation (0.9 mW/mm^2^) at 1–2.5 Hz. To highlight the FP shape only + 100 µV to −100 µV of the ~400 µV large FP is displayed. (**G**) Average FP during pacing with 1, 1.5 and to 2 Hz. (**H**) Rate-dependence of the FPD (n = 28–37 from 5 batches, *p < 0.05, ***p < 0.001, 1-Way ANOVA and Dunnett’s post testing with 1 Hz as control).

In contrast, optogenetic pacing by illuminating all recording electrodes simultaneously activated all cardiomyocytes at once without conduction delay (Fig. 3A) and in consequence FP occurred at the same time on all electrodes (Fig. 3B). Thus the ~30% ChR2 non-expressing cardiomyocytes did not cause any delay in local activation because of their equal distribution. The simultaneous activation allowed unbiased averaging of the optogenetically paced FP from all electrodes, which overall equalized the well-known variability in FP shape from individual electrodes. In fact, we found that this automatic electrode averaging very consistently resulted in a FP signal with a sharp non-fragmented initial fast FP component and a prominent, well-detectable maximal value of the repolarization wave (t_end_) (Fig. 3C).

Illuminations with pulses as short as 1 ms were sufficient for optical pacing with an average light intensity threshold for stable pacing of 0.30 ± 0.04 mW/mm^2^ (Fig. 3D). Optical pacing was very robust with low variance between individual experiments and cell batches possibly because the percentage of ChR2 expression rate was always far above threshold, which we have proven earlier in mouse hearts to be in the range of 40% of expressing cardiomyocytes^38^. Using longer pulse durations decreased the required light intensity (Fig. 3D). To exclude that photons applied to the titanium-nitride electrodes of the MEA could generate recording artefacts by photoelectric effects that have been observed during optical stimulation of field potentials in the brain^44^, we recorded electrical signals without cells. Application of supramaximal light pulses (1 ms, 0.9 mW/mm^2^) did not show any electrical signal (Fig. 3E) and even 10 ms long light pulses of 2.7 mW/mm^2^ induced only very small (<10 µV) potential deflections (Fig. 3E).

Thus we used for all subsequent experiments supramaximal stimulation (0.9 mW/mm^2^) with 1 ms long light pulses. This enabled reliable pacing of hiPSC-derived cardiomyocytes up to 2.5 Hz (=150 bpm) (Fig. 3F, Supplementary Fig. 1). Interestingly, we found decreasing FPD at higher pacing rates (Fig. 3G) with significant shorter values at 2 and 2.5 Hz compared to 1 Hz (Fig. 3H).

### Frequency-dependent drug effects on FPD

**Figure 4:**
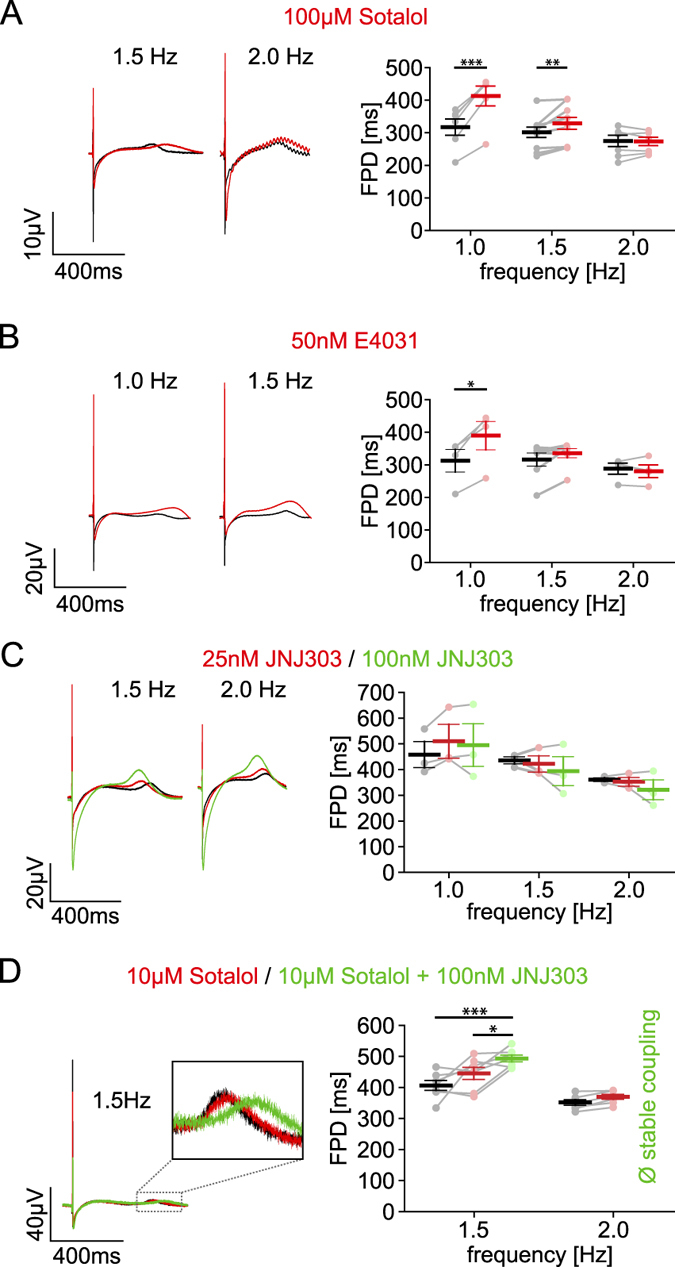
Rate-dependent effects of potassium channel blockers. (**A**–**D**) Representative averaged FP (left) and FPD values (right) in control conditions (black) and after drug application (red). (**A**) Effects of 100 µM Sotalol on FPD at 1 Hz (+30.4 ± 3.5%, n = 6 from 4 batches, p = 0.0003), 1.5 Hz (+9.1 ± 2.4%; n = 10 from 4 batches; p = 0.006) and 2 Hz (+0.1 ± 2.3%, n = 7 from 3 batches, p = 0.76), (**B**) Effect of 50 nM E4031 at 1 Hz (+24.6 ± 3.7%, n = 4 from 3 batches, p = 0.013), 1.5 Hz (+7.5 ± 4.0%, n = 7 from 3 batches, p = 0.1) and 2 Hz (−2.8 ± 3.1%, n = 4 from 3 batches, p = 0.4542). (**C**) Effect of 25 nM (red) JNJ303 in FPD at 1 Hz (+10.9 ± 2.5%, n = 3 from 2 batches, p = 0.105), 1.5 Hz (−3.3 ± 4.2%, n = 3 from 2 batches, p = 0.614), 2 Hz (−2.4 ± 2.4%, n = 3 from 2 batches, p = 0.508) and of 100 nM (green) JNJ303 at 1 Hz (+6.7 ± 5.2%, n = 3 from 2 batches, p = 0.381), 1.5 Hz (−10.2 ± 8.4%, n = 3 from 2 batches, p = 0.442) and 2 Hz (−11.3 ± 7.3%, n = 3 from 2 batches, p = 0.341). (**D**) Effect of low-dose 10 µM Sotalol at 1.5 Hz on FPD (red, + 10.6 ± 7.0, n = 7 from 1 batch, p = 0.17) and additional 100 nM JNJ303 (green, compared to control: + 22.1 ± 3.3%, p < 0.0001; compared to Sotalol: + 11.8 ± 4.3%, p = 0.03; n = 7 from 1 batch). Note that stable pacing >1.5 Hz was not possible (n = 7) after applying JNJ303 and Sotalol. ﻿Significances were determined using two-tailed, paired student’s *t*-tests. *p < 0.05, ﻿**p < 0.0﻿﻿1, ***p < 0.001.﻿

The value of our newly established optogenetic pacing of hiPSC-derived cardiomyocytes for heart rate-dependent drug screening was exemplarily tested using well-documented drugs that affect cardiac K^+^, Ca^2+^ and Na^+^ channels. It has been shown before that block of hERG channels^4^ increases FPD in MEA recordings of spontaneous beating chicken- and hiPSC-derived cardiomyocytes^1, 6, 10, 20, 24^. To analyse the rate-dependency of this effect, we tested the β-blocker Sotalol that is known to also inhibit hERG channels and prolong the QT duration in patients^45^. We found that Sotalol (100 µM) significantly increased FPD at 1 Hz pacing resembling the heart rate at rest (Fig. 4A). Interestingly this effect was less at higher rates and completely abrogated at 2 Hz pacing (Fig. 4A). To verify that this rate-dependence can be attributed to hERG channels, we used E4031, a highly specific I_Kr_ blocker at nanomolar concentrations^46^. Consistently, application of E4031 (50 nM) significantly increased FPD only at 1 Hz but not at higher frequencies (Fig. 4B). We also tried to dissect the influence of the I_Ks_ currents on the FP by application of the specific inhibitor JNJ303^1, 47^ in two different concentrations but could not detect any effect on the FPD at all pacing rates (Fig. 4C). Because of recent reports detecting I_Ks_ currents within the FP only after reducing the repolarisation reserve^1^, we similarly applied low dose (10 µM) of Sotalol to preblock hERG channels. This had only a minor effect on FPD but importantly, in this condition, we found a significant prolongation of FPD by 100 nM JNJ303 proving the functional expression of I_Ks_ channels (Fig. 4D). Interestingly, in the presence of JNJ303 and low dose Sotalol, cells could not be paced at rates higher than 1.5 Hz. This effect was not observed using Sotalol alone even at 100 µM (Fig. 4A) and it points to the importance of I_Ks_ for repolarization at higher rates. This is well in line with the clinical observation that lack of I_Ks_ channels due to inherited LQTS1 mutations are most dangerous after adrenergic stimulation and at high beating rates^48, 49^.

**Figure 5:**
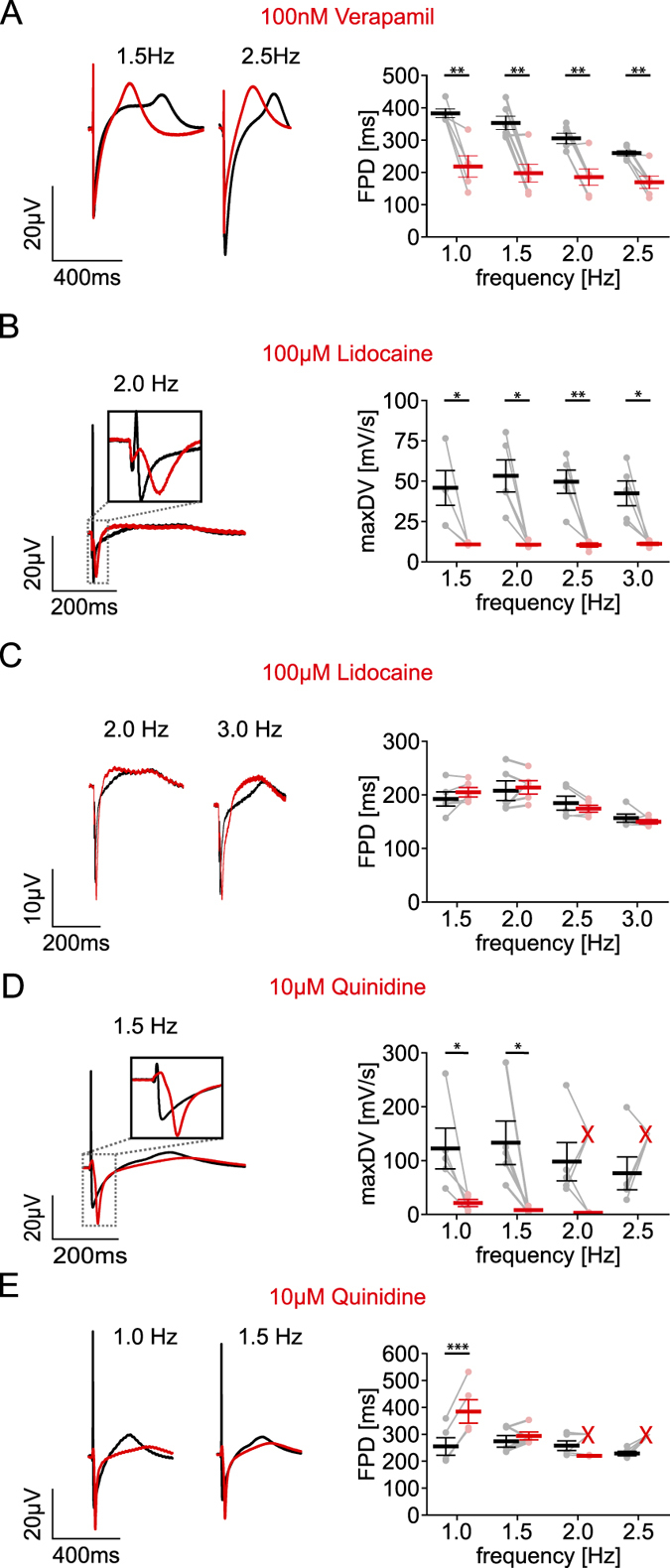
Rate-dependent effects of sodium and calcium channel blockers. (**A**–**E**) Representative averaged FP (left) and FPD values (right) in control conditions (black) and after drug application (red) (**A**) Influence of 100 nM Verapamil on the FPD at 1 Hz (−42.8 ± 8.9%, n = 5 from 3 batches, p = 0.0087), 1.5 Hz (−43.3 ± 9.0%, n = 6 from 3 batches, p = 0.0052), 2 Hz (−39.0 ± 8.6%, n = 6 from 3 batches, p = 0.0056) and 2.5 Hz (−34.6 ± 7.1%, n = 6 from 3 batches, p = 0.0045). (**B**) Influence of 100 µM Lidocaine on maximum downstroke velocity (maxDV, 1.5 Hz: −69.7 ± 6.6%, p = 0.032; 2 Hz: −76.6 ± 4.4%, p = 0.014; 2.5 Hz: −75.1 ± 6.3%, p = 0.008 and 3 Hz: −68.5 ± 6.8%, p = 0.019; all n = 5 from 1 batch). (**C**) Influence of 100 µM Lidocaine on the FPD at 1.5 Hz (+7.5 ± 4.4%, p = 0.184), 2 Hz (+4.4 ± 5.2%, p = 0.614), 2.5 Hz (−4.5 ± 3.1%, p = 0.241) and 3 Hz (−3.8 ± 3.5%, p = 0.37; all n = 5 from 1 batch). (**D**) Influence of 10 µM Quinidine on maximum downstroke velocity (maxDV, 1 Hz: −80.4 ± 6.5%, n = 5 from 3 batches, p = 0.04; 1.5 Hz: −92.5 ± 2.5%, n = 5 from 2 batches, p = 0.031). (**E**) Effect of 10 µM Quinidine on FPD at 1 Hz (+51.8 ± 2.8%, n = 5 from 3 batches, p = 0.0004) and 1.5 Hz (+8.8 ± 5.9%, n = 5 from 2 batches, p = 0.29). In the presence of Quinidine some MEA experiments could not be paced >2 Hz indicated by a red “X” (**B**,**C**). Significances were determined using two-tailed, paired student’s *t*-tests. *p < 0.05, ﻿**p < 0.0﻿﻿1, ***p < 0.001.

To prove the ability to detect drug-induced SQT syndromes, we inhibited Ca^2+^ channels, which should shorten the APD. We found that application of the specific L-type Ca^2+^ channel blocker Verapamil (100 nM) significantly decreased FPD at all pacing frequencies (Fig. 5A). Interestingly, the intrinsic rate-dependency of FPD was less evident in the presence of Verapamil (21.3 ± 2.0% shorter FPD at 2.5 Hz compared to 1 Hz) than without Verapamil (31.2 ± 1.8% shorter FPD at 2.5 Hz compared to 1 Hz, n = 5 from 3 batches, p = 0.0493, paired student’s *t*-test) indicating a potential involvement of Ca^2+^ channels in this effect.

### Determining Na^+^ channel availability

Finally we tested drugs leading to BS which can be caused by reduced activity of Na^+^ channels leading to slowing of AP upstroke and conduction velocity, and results in a high risk for lethal arrhythmia^50, 51^. To test whether such drug effects can be measured using optogenetically paced FP, we analysed the maximum downstroke velocity during the fast initial component of the FP, which is thought to reflect the AP upstroke^21^. In fact, this value was significantly reduced by application of the selective Na^+^ channel blocker Lidocaine (100 µM) at all beating rates from 1.5 to 3 Hz (Fig. 5B), whereas the FPD was unaffected (Fig. 5C). This principle was further confirmed by testing Quinidine (10 µM) which decreased the maximum downstroke velocity both at 1 Hz and 1.5 Hz (Fig. 5D). Interestingly, Quinidine also decreased the spontaneous beating rate (−39.0 ± 1.2%, n = 6 from 3 batches, p =  <0.001) and hindered pacing at high rates (successful pacing of MEA: 40% at 2 Hz and 0% at 2.5 Hz, n = 5 from 2 batches, Fig. 5D and E) suggesting a reduced excitability of cardiomyocytes. Because it is known that Quinidine also affects hERG channels^6^, we analysed FPD and found increased values at 1 Hz but not at higher frequencies (Fig. 5E) which is in line with the rate-dependency of hERG block by Sotalol and E4031.

### Magnetic field targeting of cardiomyocytes

**Figure 6:**
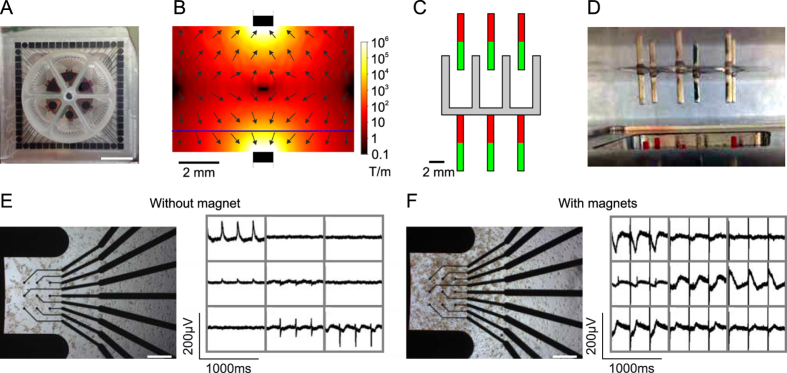
Magnetic cardiomyocytes positioning on MEA electrodes. (**A**) Top view of a 6-well multi-well MEA. Scale bar: 1 cm. (**B**) Absolute value (colour) and direction (arrows) of magnetic flux density gradient with two magnets in a cut through the symmetry plane (blue line indicates the level of the recording electrodes). (**C**,**D**) Schematic drawing (**C**) and side view (**D**) of the magnet setup. (**E**,**F**) Bright field image (left) and FP from the 9 electrodes (right) of a well after plating 1 × 10^4^ MNP loaded hiPSC-derived cardiomyocytes without magnets (**E**) or within the magnet setup (**F**). Scale bars: 400 µm (**E**,**F**).

Because hiPSC-derived cardiomyocytes are cost-intensive and confluent monolayers are required for good FP signals, we used manual drop-seeding of cells on the field of electrodes after local coating with fibronectin. However for future high throughput MEA recordings, automatized cell seeding is mandatory. Therefore we established magnetic cardiomyocyte positioning on multi-well MEA chips with 9 recording electrodes per well (Fig. 6A). To achieve this, cylindrical rare-earth magnets were used and their position was optimized by simulating the magnetic flux density and its gradient (Fig. 6B) until a maximum focused magnetic gradient was reached at the level of the recording electrodes. We found an optimal configuration by placing a 1 mm diameter magnet directly below the 1 mm thick MEA glass substrate and a second magnet 5 mm above (Fig. 6C,D). Thereby we obtained a maximum magnetic gradient of ~120 T/m at the centre of the well with a full width at half maximum of ~2 mm. To test this magnet setup, hiPSC derived cardiomyocytes were loaded with the magnetic nanoparticle SO-Mag5^39, 41^ overnight and only 1 × 10^4^ MNP-loaded cells were applied to each well of the multi-well MEA. Without a magnet this cell number resulted in sparsely distributed cardiomyocytes and only few electrodes showed small FP (Fig. 6E). However when placing the MEA chip in the newly designed magnetic setup (Fig. 6D) for 12 h hours after cell seeding, we found a monolayer of cardiomyocytes attached to the recording electrodes resulting in high amplitude FP (Fig. 6F).

## Discussion

The here presented approach using optogenetic pacing of hiPSC-derived cardiomyocytes on MEA will add to the safety and effectiveness of drug screening because of five distinct technical and biological advantages over previous MEA recordings using spontaneous beating cardiomyocytes:

1. In contrast to mature ventricular cardiomyocytes in the heart, the currently available hiPSC-derived cardiomyocytes are spontaneously active because they represent a rather embryonic-like phenotype with a mixture of atrial and ventricular-like cells with pacemaker potential. However enhanced maturation and selection protocols to generate more mature ventricular-like hiPSC-derived cardiomyocytes are emerging, which will result in lower or lack of spontaneous activity^52–54^ and thus make pacing in drug screening applications indispensable. In this regard, the here presented optogenetic pacing up to 2.5 Hz will provide a straight forward determination of maximum frequency response and the precise analysis of excitation threshold, which are important parameters not only for drug action but also for cardiomyocyte maturation.
2. The simultaneous optogenetic activation of all cardiomyocytes on a MEA allows spatial averaging of the FP from all electrodes resulting reliably in one robust FP shape. This enables objective analysis which could be easily automatized in the future in contrast to FP recordings from MEA with individual electrodes which require user input for choosing the electrodes suited for analysis.
3. The lack of conduction upon global illumination yield a non-fragmented fast initial negative voltage deflection in the averaged FP, which can be used to quantify Na^+^ channel function. This is in contrast to unbiased averaging of non-synchronous FP during spontaneous beating because electrical activity is locally initiated and sequentially conducted. Importantly, this would also occur during electrical stimulation because only cells near the cathode are depolarized whereas those near the anode are hyperpolarized^55^. The precise analysis of the initial FP component also highlights the advantage of optogenetically paced MEA recordings over the recently reported all-optical cardiac toxicity screening assays using camera recordings of voltage-sensitive dyes or proteins, which is limited to frame rates below 1 kHz for sufficient signal to noise ratio^18, 19^. In contrast, MEA recordings can be performed at a sampling frequency >10 kHz, which is essential to detect variations in the very fast initial component of the FP (>100 µV/ms), reflecting the fast AP upstroke velocity of hiPSC-derived cardiomyocytes (>30 mV/ms) and therefore is well-suited to identify even slight reduction of Na^+^ channel activity.
4. In the future, the simultaneous activation of all cardiomyocytes over the large distance of 1.4 × 1.4 mm by global illumination will allow using large recording electrodes which will – similar to our automatic unbiased FP averaging – physically average the FP generated by thousands of cardiomyocytes. Such large electrodes are already used in scalable multi-well MEA systems^56^, which enable parallel recording of drug effects. For cell plating on such multi-well MEA, the required cell numbers could be reduced using our new method of magnetic nanoparticle guided seeding of cardiomyocytes on the recording electrodes.
5. Finally, optogenetic pacing allows analysing drug-effects over the whole physiological range of human heart rate, which is essential given the use-dependent properties of cardiac ion channels and the rate-dependency of drugs^31–33^.

In fact, we detected shortening of FPD at higher rates by recording the extracellular FP from a monolayer of hiPSC-derived cardiomyocytes. This is similar to shortening of QT duration in the human ECG at high heart rates *in vivo* and in full concordance to electrophysiological recordings and mathematical simulations of human cardiomyocytes^2^ as well as single cell recordings of hiPSC - derived cardiomyocytes^54^. Interestingly, the intrinsic high rate-induced FPD shortening was diminished when blocking Ca^2+^ channels with verapamil, suggesting that refractoriness or Ca^2+^ accumulation-dependent inactivation of Ca^2+^ channels is contributing to the APD rate dependency in hiPSC-derived cardiomyocytes. The remaining frequency dependence of APD even when Ca^2+^ channels were blocked could be explained by Na^+^ accumulation at high rates with secondary augmentation of Na^+^/K^+^ exchanger current as suggested by the O’Hara/Rudy model of human ventricular myocytes^2^. FP from the MEA recordings were consistently longer than APs from the patch clamp experiments, which is a previously described effect and was analysed before using simultaneous intracellular sharp electrode recordings and FP recordings on MEA^21^. Importantly the duration of FP in our study are within the same range than other reports using hiPSC-derived cardiomyocytes.

Because of the rate-dependency of AP and QT duration, several QT_c_ correction formulas were developed (e.g. Bazett or Frederica) for the human ECG and even used to correct for rate-effects of FPD in MEA recordings^22–24^. However, none of these formulas were validated for hiPSC-derived cardiomyocytes, and alterations of the intrinsic rate dependency of APD by drugs or mutations are neglected. Our results point to the possibility that the herein used hERG channel blockers Sotalol, E4031 and quinidine lead to prolongation of the FPD only at lower heart rates. This is in line with the clinical observation that slow beating rates and especially the first beat after a long post extrasystolic pause, is the most dangerous trigger for the induction of malignant torsades-de-pointes arrhythmia in drug-induced LQTS^4^.

Importantly, our approach is based on simple protocols which all can be automatized in a straight-forward manner. Gene transfer of ChR2 by AAV application can be performed parallelized by standard liquid handling robots and automatized magnetic cell positioning could be performed in commercially available multi-well MEA^56^. The required light intensity for pacing is very low and can be generated by cheap LEDs. Finally we provide evidence that the spatially averaging allows the unbiased and automated analysis of FPD and initial FP component. Thus we believe that our report will lay out the basis for an easy-to-handle all-in-one system for preclinical cardiac toxicity screening at pharmaceutical companies.

In addition to the drug screening for cardiac safety, iPSC-derived cardiomyocytes could be employed in personalized medicine for patients with rare inherited LQTS, SQTS, BS or other arrhythmia, even for those with a known phenotype but unknown or uncertain causal mutation. In such individuals, therapy is often based on trial and error with off-label use of drugs because evidence-based drug testing in clinical studies is not feasible due to the low patient numbers and high risk of lethal arrhythmia. *In vitro* drug screening using cardiomyocytes from patient-specific iPSC can be performed with unlimited number of different compounds and will intrinsically take the genetic context and unknown modifiers of the phenotype into account^57, 58^. Importantly, the use of AAV for ChR2 gene transfer represents a straight forward and easy to use method to be applied to all existing patient-specific hiPSC cell lines without the need for lengthy generation and validation of new ChR2 transgenic iPSC lines and the risk of epigenetic changes in the subclones. Analysing drug action over the whole physiological heart rate using optogenetic pacing is essential given the intrinsic frequency-dependent effects of mutations. For instance, LQTS1 patients with loss-of-function mutations in the genes encoding for the I_Ks_-channels are prone to arrhythmias only at high beating rates and LQTS3-causing gain-of-function mutations exert their effects only at slow rates leading to arrhythmia and sudden cardiac death at rest or during sleep^31^.

## Electronic supplementary material

Supplementary Figure 1
