# Author Correction: Frequency-dependent drug screening using optogenetic stimulation of human iPSC-derived cardiomyocytes

**DOI:** 10.1038/s41598-020-80763-7

**Published:** 2021-01-12

**Authors:** Hendrik Lapp, Tobias Bruegmann, Daniela Malan, Stephanie Friedrichs, Carsten Kilgus, Alexandra Heidsieck, Philipp Sasse

**Affiliations:** 1 Institute of Physiology I, Life and Brain Center, Medical Faculty, University of Bonn, Sigmund-Freud-Str. 25, 53127 Bonn, Germany; 2 Research Training Group 1873, University of Bonn, 53127 Bonn, Germany; 3 Zentralinstitut für Medizintechnik, Technische Universität München, München, Germany

Correction to: *Scientific Reports* 10.1038/s41598-017-09760-7, published online 29 August 2017

This Article contains an error in the Experimental Procedures section under subheading ‘Cell culture and generation of ChR2-expressing cardiomyocytes’, where data is omitted in the first paragraph,

“1 × 10^6^ Cor4U cardiomyocytes derived from hiPSC were purchased from Axiogenesis (Cologne, Germany) and cultivated in Cor4U Complete Culture Medium (Axiogenesis) in T25 flasks. This cell line has been previously characterized in detail^17,35,36^ and consists of ventricular (~60%), atrial (~20%) and pacemaker-like (20%) cardiomyocytes^37^. For expression of ChR2, Cor4U cardiomyocytes were transduced in the T25 flask with an adeno-associated virus (AAV) at 6.6 × 10^4^ genome copy numbers per cell.

should read:

“1 × 10^6^ Cor4U cardiomyocytes derived from hiPSC were purchased from Axiogenesis (Cologne, Germany) and cultivated in Cor4U Complete Culture Medium (Axiogenesis) in T25 flasks. This cell line has been previously characterized in detail^17,35,36^ and consists of ventricular (~60%), atrial (~20%) and pacemaker-like (20%) cardiomyocytes^37^. After studies had been completed short tandem repeat testing by Axiogenesis/NCardia determined that Cor.4U cardiomyocytes were derived from the human embryonic stem cell line RUES2. The different provenience of these well characterized cardiomyocytes does neither change the findings nor the interpretation of the data. Thus the term “iPSC” used in this paper refers to “pluripotent stem cells”. For expression of ChR2, Cor4U cardiomyocytes were transduced in the T25 flask with an adeno-associated virus (AAV) at 6.6 × 10^4^ genome copy numbers per cell.

