# Supplementary material for: Frequency-dependent drug screening using optogenetic stimulation of human iPSC-derived cardiomyocytes
Source: Sci Rep. 2017 Aug 29;7:9629. doi: 10.1038/s41598-017-09760-7 (PMC5575076; doi:10.1038/s41598-017-09760-7)
Supplement: Supplementary file 1 — Supplementary Figure 1 [file 41598_2017_9760_MOESM1_ESM.pdf]

Supplementary Information

Frequency-dependent drug screening using optogenetic stimulation of human iPSC-derived cardiomyocytes

Hendrik Lapp[1,#], Tobias Bruegmann[1,2,#], Daniela Malan[1], Stephanie Friedrichs[1], Carsten Kilgus[1], Alexandra Heidsieck[3], Philipp Sasse[1]*

[1] Institute of Physiology I, Life and Brain Center, Medical Faculty, University of Bonn, Sigmund-Freud-Str. 25, 53127 Bonn, Germany
[2] Research Training Group 1873, University of Bonn, 53127 Bonn, Germany
[3] Zentralinstitut für Medizintechnik, Technische Universität München, München, Germany

[#] These authors contributed equally to this work.

* Corresponding author

Supplementary Figure 1

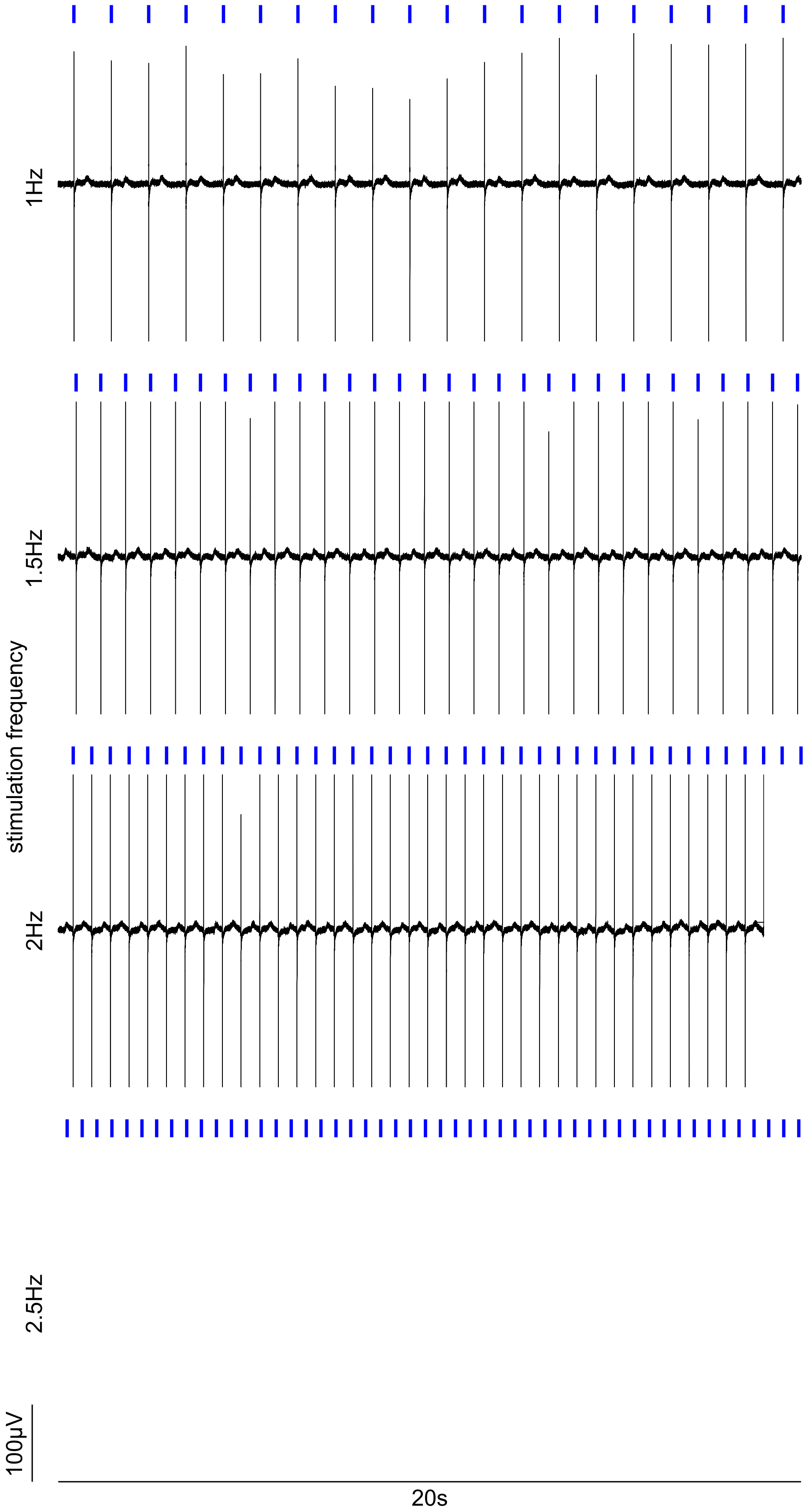

Supplementary Figure 1 Optical stimulation of cardiomyocytes. Representative FP recordings over the whole recording time of 20 seconds from one electrode upon optical stimulation (0.9 mW/mm^2) at 1-2.5 Hz. To highlight the FP shape only +100 µV to -100 µV of the ~400µV large FP is displayed.
